# Comparison of the Relative Diagnostic Performance of [^68^Ga]Ga-DOTA-FAPI-04 and [^18^F]FDG PET/CT for the Detection of Bone Metastasis in Patients With Different Cancers

**DOI:** 10.3389/fonc.2021.737827

**Published:** 2021-09-17

**Authors:** Junhao Wu, Yingwei Wang, Taiping Liao, Zijuan Rao, Weidong Gong, Lei Ou, Yue Chen, Chunyin Zhang

**Affiliations:** ^1^Department of Nuclear Medicine, The Affiliated Hospital, Southwest Medical University, Luzhou, China; ^2^Nuclear Medicine and Molecular Imaging Key Laboratory of Sichuan Province, Luzhou, China; ^3^Academician (Expert) Workstation of Sichuan Province, Luzhou, China

**Keywords:** bone metastases, [^68^Ga]Ga-DOTA-FAPI-04, [^18^F]FDG, PET/CT, cancer

## Abstract

**Purpose:**

The present retrospective analysis sought to compare the relative diagnostic efficacy of [^68^Ga]Ga-DOTA-FAPI-04 to that of [^18^F]FDG PET/CT as a means of detecting bone metastases in patients with a range of cancer types.

**Materials:**

In total, 30 patients with bone metastases associated with different underlying malignancies were retrospectively enrolled. All patients had undergone [^68^Ga]Ga-DOTA-FAPI-04 and [^18^F]FDG PET/CT, and the McNemar test was used to compare the relative diagnostic performance of these two imaging modalities. The maximum standard uptake value (SUVmax) was used to quantify radiotracer uptake by metastatic lesions, with the relative uptake associated with these two imaging strategies being compared *via* the Mann-Whitney U test. The cohort was further respectively divided into two (osteolytic and osteoblastic bone metastases) and three clinical subgroups (lung cancer, thyroid cancer, and liver cancer).

**Results:**

[^68^Ga]Ga-DOTA-FAPI-04 PET/CT was found to be significantly more sensitive as a means of diagnosing bone metastases relative to [^18^F]FDG PET/CT ([109/109] 100% *vs* [89/109] 81.7%; P< 0.01), consistent with the significantly increased uptake of [^68^Ga]Ga-DOTA-FAPI-04 by these metastatic lesions relative to that of [^18^F]FDG (n=109, median SUVmax, 9.1 *vs*. 4.5; P< 0.01). [^68^Ga]Ga-DOTA-FAPI-04 accumulation was significantly higher than that of [^18^F]FDG in both osteolytic (n=66, median SUVmax, 10.6 *vs* 6.1; P < 0.01), and osteoblastic metastases (n=43, median SUVmax, 7.7 *vs* 3.7; P < 0.01). [^68^Ga]Ga-DOTA-FAPI-04 uptakes were significantly higher than that of [^18^F]FDG in bone metastases from lung cancer (n = 62, median SUVmax, 10.7 *vs* 5.2; P < 0.01), thyroid cancer (n = 18, median SUVmax, 5.65 *vs* 2.1; P < 0.01) and liver cancer (n = 12, median SUVmax, 5.65 *vs* 3.05; P < 0.01). However, [^68^Ga]Ga-DOTA-FAPI-04 detected 10 false-positive lesions, while only 5 false-positive were visualized by [^18^F]FDG PET/CT.

**Conclusion:**

[^68^Ga]Ga-DOTA-FAPI-04 PET/CT exhibits excellent diagnostic performance as a means of detecting bone metastases, and is superior to [^18^F]FDG PET/CT in this diagnostic context. Furthermore, [^68^Ga]Ga-DOTA-FAPI-04 tracer uptake levels are higher than those of [^18^F]FDG for most bone metastases. However, owing to the potential for false-positive bone lesions, it is critical that physicians interpret all CT findings with caution to ensure diagnostic accuracy.

## Introduction

Many cancers, including prostate and breast cancer, frequently metastasize to the bone, resulting in a marked rise in the difficulty of treating these diseases and concomitant increases in patient mortality (1–5). An estimated 350,000 individuals die while suffering from malignant bone metastases in the United States each year (4). While such metastases are rarely a direct cause of death, they can lead to the development of a range of complications such as acute pain, pathological bone fractures, spinal paralysis, and hypercalcemia, all of which can compromise patient quality of life and contribute to a poor prognosis (1, 3, 4, 6, 7). Detecting such metastases in a timely manner is thus critical to ensure accurate disease staging and to guide the selection of appropriate management strategies aimed at improving comfort and survival.

Clinical tumor staging is an important process that can directly impact patient prognostic evaluation and treatment planning. Bone scintigraphy (BS) is among the most common approaches used to test for malignant bone metastases, owing to its low cost and amenability to whole-body examinations (8–10). However, the relative insensitivity for detecting changes in tumor viability by indirect measurement of osteoblastic activity, making it of limited value when monitoring therapeutic responses in patients. In addition, certain benign lesions can yield a false-positive signal upon BS evaluation, limiting the specificity of this imaging technique (10, 11). [^18^F]NaF is a specific bone imaging tracer that is pharmacologically similar to BS, and prior evidence suggests that [^18^F]NaF PET/CT scans are superior to BS for bone metastasis detection (1, 9, 12, 13). However, [^18^F]NaF uptake can similarly be enhanced by non-malignant bone diseases, further limiting the diagnostic specificity of this imaging modality (1, 12).

In recent years, [^18^F]Deoxyglucose (FDG) PET/CT has been applied as a tool for evaluating suspicious bone lesions owing to its ability to detect more lesions relative to traditional BS, with fused PET/CT images allowing clinicians to view these lesions in their surrounding anatomical context (10, 14–16). [^18^F]FDG PET/CT has been shown to exhibit high sensitivity and specificity in bone marrow, osteolytic, and mixed metastases, underscoring the advantageous nature of this technique (10, 15). However, abnormal uptake of the [^18^F]FDG radiotracer in bone can nonetheless occur as a consequence of hematopoietic cytokine stimulation, infection, fractures, benign bone lesions, and benign hematological diseases, somewhat limiting the ability of this approach to detect bone metastases (17–19).

Owing to the limitations associated with extant imaging modalities used to detect bone metastases, there is a clear need for the development of novel approaches. Tumor growth is dependent not only on the underlying cancer cells, but also on the properties of certain non-malignant tumor-associated stromal cells, which can drive tumorigenicity (20). Cancer-associated fibroblasts (CAFs) are important components of the stromal compartment, and can alter microenvironmental characteristics in a manner that can be conducive to bone metastasis (21). Indeed, CAFs are often associated with a poor cancer patient prognosis. In many malignant contexts, CAFs express high levels of fibroblast activation protein (FAP) (22–25). A novel PET tracer based upon quinoline FAP-specific inhibitors (FAPI) has recently been developed and used to target FAP and to visualize the tumor stroma (26–29). Relative to [^18^F]FDG, [^68^Ga]Ga-DOTA-FAPI-04 exhibits high uptake by tumors, is associated with a lower level of background signal, and exhibits good pharmacokinetic properties *in vivo*, making it ideal for contrast and visibility (29, 30). As such, [^68^Ga]Ga-DOTA-FAPI-04 PET/CT has emerged as an imaging approach for evaluating malignant tumors and associated metastatic lesions. This approach has recently been reported to be effective for the detection of bone metastases and to be superior to [^18^F]FDG PET/CT with respect to diagnostic utility in this context (23, 28, 29, 31–33). However, there have been few systematic studies conducted to date comparing the diagnostic efficacy of [^68^Ga]Ga-DOTA-FAPI-04 and [^18^F]FDG PET/CT as tools for detecting bone metastases in patients with various cancers. Relative to [^18^F]FDG PET/CT, the clinical utility of a similar imaging strategy instead using [^68^Ga]Ga-DOTA-FAPI-04 as a radiotracer for the detection of malignant bone metastases remains to be established. As such, we herein performed a comparative analysis of the relative performance of [^68^Ga]Ga-DOTA-FAPI-04 and [^18^F]FDG PET/CT for the detection of bone metastases in patients with a range of tumor types, working under the hypothesis that [^68^Ga]Ga-DOTA-FAPI-04 is superior to [^18^F]FDG PET/CT in this diagnostic context.

## Patients, Materials and Methods

We retrospectively studied 30 patients (18 male, 12 female) with advanced cancer complicated by bone metastases who had undergone both [^68^Ga]Ga-DOTA-FAPI-04 and [^18^F]FDG PET/CT at the Affiliated Hospital of Southwest Medical University from February 2020 - September 2020. The inclusion criteria were: (i) [^68^Ga]Ga-DOTA-FAPI-04 and [^18^F]FDG PET/CT were performed within 7 days; (ii) None of the skeletal metastatic lesions had received treatment before imaging analyses; (iii) no history of other primary malignancies. The exclusion criteria were: (i) PET or bone scan results were not available; (ii) Patients who were lost to follow-up; (iii) No definite histopathological diagnosis of the primary lesion.

22 patients had not received any previous treatment for their primary and metastatic tumors. 7 patients received surgical resection of the primary tumors. In addition, 1 patient received interventional treatment and local radiotherapy of the primary liver cancer. For an overview of patient characteristics, see **Table 1**.

**Table 1:** Characteristics of the 30 patients with bone metastases.

Total patients					N = 30
Age (years)					
Median					58.4 ± 13.8
Range					25 - 78
Sex					
Male					18 (60%)
Female					12 (40%)
Inspection purpose					
Newly diagnosed					22 (73.3%)
Suspected recurrence or progression					8 (26.7%)
Time interval (days)					
Median					2.6 ± 1.9
Range					1 - 7
Pathological types					
Lung cancer					11 (36.7%)
Thyroid cancer					5 (16.7%)
Liver cancer					5 (16.7%)
Prostate cancer					3 (10.0%)
Breast cancer					1 (3.3%)
Nasopharyngeal cancer					1 (3.3%)
Cervical cancer					1 (3.3%)
Ovarian cancer					1 (3.3%)
Renal cancer					1 (3.3%)
Pancreatic cancer					1 (3.3%)

N, number.

All patients were followed for a minimum of 6 months (9.2 ± 2.3 mo; 6-14 mo). Biopsy-based confirmation of patient bone metastases was not conducted for ethical and practical reasons, with the final diagnosis of these metastases instead being based upon a combination of imaging examination results (BS, CT, MRI, or PET/CT) and clinical follow-up (physical signs and follow-up imaging examination).

This study was approved by the Ethics Committee of the First Affiliated Hospital of Southwest Medical University and followed the 1964 Helsinki Declaration and its subsequent amendments to the ethical standards. All patients signed written informed consent forms.

### Preparation of [^18^F]FDG and [^68^Ga]Ga-DOTA-FAPI-04

[^18^F]FDG was prepared using the Siemens Eclipse HD cyclotron and [^18^F]FDG automated chemical synthesis system.

The precursor FAPI-04 from MCE (MedChemExpress, USA) with a purity grade of 98% and a mass of 872.91. FAPI-04 radiolabeling was performed according to the following protocol: 50 μg of FAPI-04 was dissolved in 1 mL of sodium acetate solution (0.25 M) and added 4 mL ^68^Ga-solution (1.7 GBq) to a pH of 3.3–3.6. The reaction was heated at 80°C for 10 min and the product was purified by using a Sep-pak ^18^C column. It was then eluted with 1 mL of 50% ethanol and 4 mL of saline. Quality control was performed by Radio-HPLC on an ^18^C reverse phase column with a gradient elution of either H_2_O with 0.1% TFA (solvent A) or CH_3_CN with 0.1% TFA (solvent B). The mobile phase conditions were 0–50 min: 10–90% B, 1 mL/min.

The [^18^F]FDG and [^68^Ga]Ga-DOTA-FAPI-04 have both radiochemical purity of > 95%, and the final product is sterile and pyrogen-free.

### PET/CT Imaging

Patients are required to avoid strenuous exercise and fast for at least 4-6 hours before the [^18^F]FDG PET/CT scan. Before intravenous injection of [^18^F]FDG to ensure blood sugar levels<11.1 mmol/L. The injected activity for the [^18^F]FDG and [^68^Ga]Ga-DOTA-FAPI-04 are 3.7 MBq/kg and 1.85–2.59 MBq/kg, respectively. Imaging was performed 50–60 min after radiotracer injection. PET/CT images acquisition were scheduled to start at 1 h (FDG: 66 ± 5 min; FAPI: 65 ± 5 min) after injection. The patients underwent PET/CT scanning on a Philips Gemini TF 16 scanner after emptying the bladder. 16-slice spiral CT scan was performed, ranging from the base of the skull to the middle upper thighs, with the arms raised above the head (120 kV, 100 mA, layer thickness 0.5 mm, matrix 512 × 512 pixels, window width 300–500 HU, window level 40–60 HU). If a patient was known to have any abnormal lesions in the limbs, he/she was scanned from the top of the head to the feet, with the arms at the sides of the body. After the CT was complete, three-dimensional PET was performed for 70–90 s per bed position, for a total of 7 bed positions. The resulting images were corrected by attenuation and reconstructed iteratively using the ordered subset expectation maximization method [3 iterations, 23 subsets, image size 144 × 144 (matrix)] to obtain transverse, coronal, and sagittal views of the PET/CT scans. If there were abnormal [^18^F]FDG uptakes in the gastrointestinal tract or urinary system that is difficult to distinguish from physiological uptake, delayed imaging was performed 2 hours after the tracer agent injection. Nuclear medicine checked the patient’s general condition (mental state/blood pressure/heart-rate/body temperature) until 120 min after radiotracer injection and were required to report any abnormalities. All the above inspection procedures were communicated to patients before obtaining their written informed consent.

### Patient Image Analyses

All [^68^Ga]Ga-DOTA-FAPI-04 and [^18^F]FDG PET/CT scans were independently interpreted in a visual, semi-quantitative manner by two experienced nuclear medicine physicians. If there were disagreements, they re-evaluate the lesion by readers together. PET, CT, and fused PET/CT imaged from all patients were examined in the axial, coronal, and sagittal planes, with bone lesions being separated into eight regions (vertebrae, rib, scapula, cranial bone, pelvis, sternum, clavicle, and long bone). For visual analyses, all bone lesions exhibiting signal intensity above that of the background area were noted as bone metastases. On both [^68^Ga]Ga-DOTA-FAPI-04 and [^18^F]FDG PET/CT, up to ten identical suspicious bone metastases in each patient were identified and categorize as either osteolytic or osteoblastic bone metastases. In semi-quantitative analyses, the two physicians manually drew round regions of interest (ROIs) around suspected metastases such that lesion SUVmax values could be determined, with the highest value for each lesion being retained for semiquantitative analysis. These SUVmax values were not used in visual analyses.

### Statistical Analysis

SPSS (v 26.0; IBM, NY, USA) was used for all statistical analyses. Differences in the rate of bone metastasis detection between these two PET/CT imaging approaches were compared *via* the McNemar test, while differences in the SUVmax values of the two tested radiotracers were compared *via* the Mann-Whitney U test, as were differences in PET radiotracer accumulation in osteoblastic and osteolytic bone metastases. P < 0.05 was the significance threshold for these analyses.

## Results

In total, 30 patients were retrospectively enrolled in this study. Bone metastases were successfully detected by [^68^Ga]Ga-DOTA-FAPI-04 PET/CT imaging in all 30 patients, whereas such metastases were detected in just 26 patients *via* [^18^F]FDG PET/CT. For a single patient, the sensitivity of [^68^Ga]Ga-DOTA-FAPI-04 is higher than that of [^18^F]FDG PET/CT, while there is no statistical difference between the two (100% [30/30] *vs* 86.7% [26/30], P = 0.125). A total of 23 primary lesions were detected. For the detection of primary lesions, the sensitivity of [^68^Ga]Ga-DOTA-FAPI-04 PET/CT was 100% (23/23), compared with 95.7% (22/23) for [^18^F]FDG PET/CT (P>0.99). 1 primary lesion of renal cancer was false-negative on [^18^F]FDG PET/CT.

In total, 109 bone metastases were confirmed based on imaging examination results (BS, CT, MRI, or PET/CT) and clinical follow-up (physical signs and follow-up imaging examination). In 14 patients (81 bone metastatic lesions), the diagnosis of bone metastasis was based on typical performance (extensive bone metastases throughout the skeleton) on PET/CT and corresponding characteristic morphologic findings of metastasis on the CT component. In 16 patients (28 bone metastatic lesions), bone metastases were judged on the findings of improvement or progression of bone metastatic lesions following treatment at follow-up imaging examination results. 10 lesions were categorized as benign lesions owing to typical appearance on imaging examination (CT or MRI) and no progressive performance was found during the follow-up period. 119 bone lesions were found to have increased FAPI uptakes. Among these FAPI-avid lesions, 109 bone lesions were considered to represent true positive lesions of bone metastases, while ten benign lesions of false-positive uptakes were identified. 94 bone lesions showed increased tracer activities on [^18^F]FDG PET/CT. As compared to [^68^Ga]Ga-DOTA-FAPI-04 PET/CT, just 89 bone metastases (81.7% of FAPI-positive bone metastatic lesions) being identified on [^18^F]FDG PET/CT. Five benign lesions of ten FAPI-avid lesions (50% of FAPI-positive benign lesions) were also detected upon [^18^F]FDG PET/CT imaging.

The most common sites of bone metastases in analyzed patients were, in order, the vertebrae (50/109), rib (19/109), pelvis (16/109), cranial bone (10/109), long bone (5/109), scapula (4/109), sternum (3/109), clavicle (2/109). Bone metastases in all lesions were successfully detected by [^68^Ga]Ga-DOTA-FAPI-04 PET/CT, while rates of detection for [^18^F]FDG PET/CT were, in order from highest to lowest: scapula [100% (4/4)], sternum [100% (3/3)], clavicle [100% (2/2)], pelvis [88% (14/16)], vertebrae [86% (43/50)], long bone [80% (4/5)], rib [74% (14/19)], cranial bone [50% (5/10)]. For further details regarding the regional detection rates associated with these two imaging strategies, see **Table 2**.

**Table 2:** Regions of bone metastases detected by [^68^Ga]Ga-DOTA-FAPI-04 and [^18^F]FDG PET/CT.

Region-based		Sensitivity (%)	
		FDG	FAPI
Vertebrae		86(43/50)	100 (50/50)
Rib		74 (14/19)	100 (19/19)
Scapula		100 (4/4)	100 (4/4)
Cranial bone		50 (5/10)	100 (10/10)
Pelvis		88 (14/16)	100 (16/16)
Sternum		100 (3/3)	100 (3/3)
Clavicle		100 (2/2)	100 (2/2)
Long bone		80 (4/5)	100 (5/5)
Total		82 (89/109)	100 (109/109)

**Figure 1:**
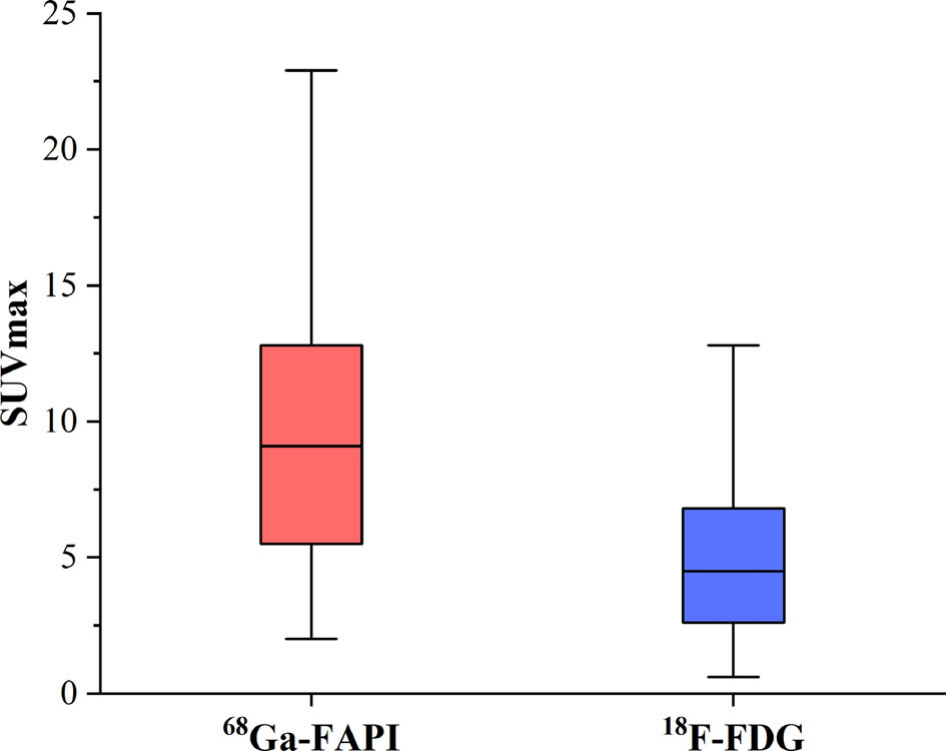
The SUVmax of FAPI and FDG in 77 bone metastatic lesions. The tracer accumulation of [^68^Ga]Ga-FAPI-04 in bone metastases is significantly higher than that of [^18^F]FDG (n=109, median SUVmax, 9.1 *vs* 4.5; P< 0.01, respectively).

**Figure 2:**
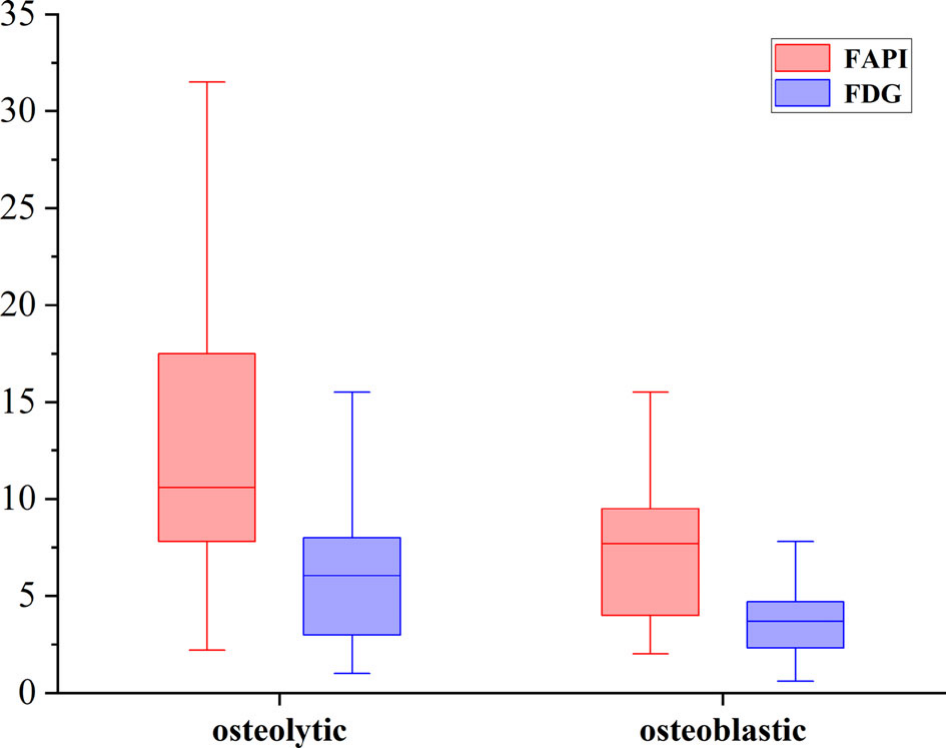
The SUVmax of FAPI and FDG in osteolytic and osteoblastic lesions. [^68^Ga]Ga-DOTA-FAPI-04 accumulation was significantly higher than that of [^18^F]FDG in both osteolytic (n=66, median SUVmax, 10.6 *vs* 6.1; P < 0.01), and osteoblastic metastases (n=43, median SUVmax, 7.7 *vs* 3.7; P < 0.01).

**Figure 3:**
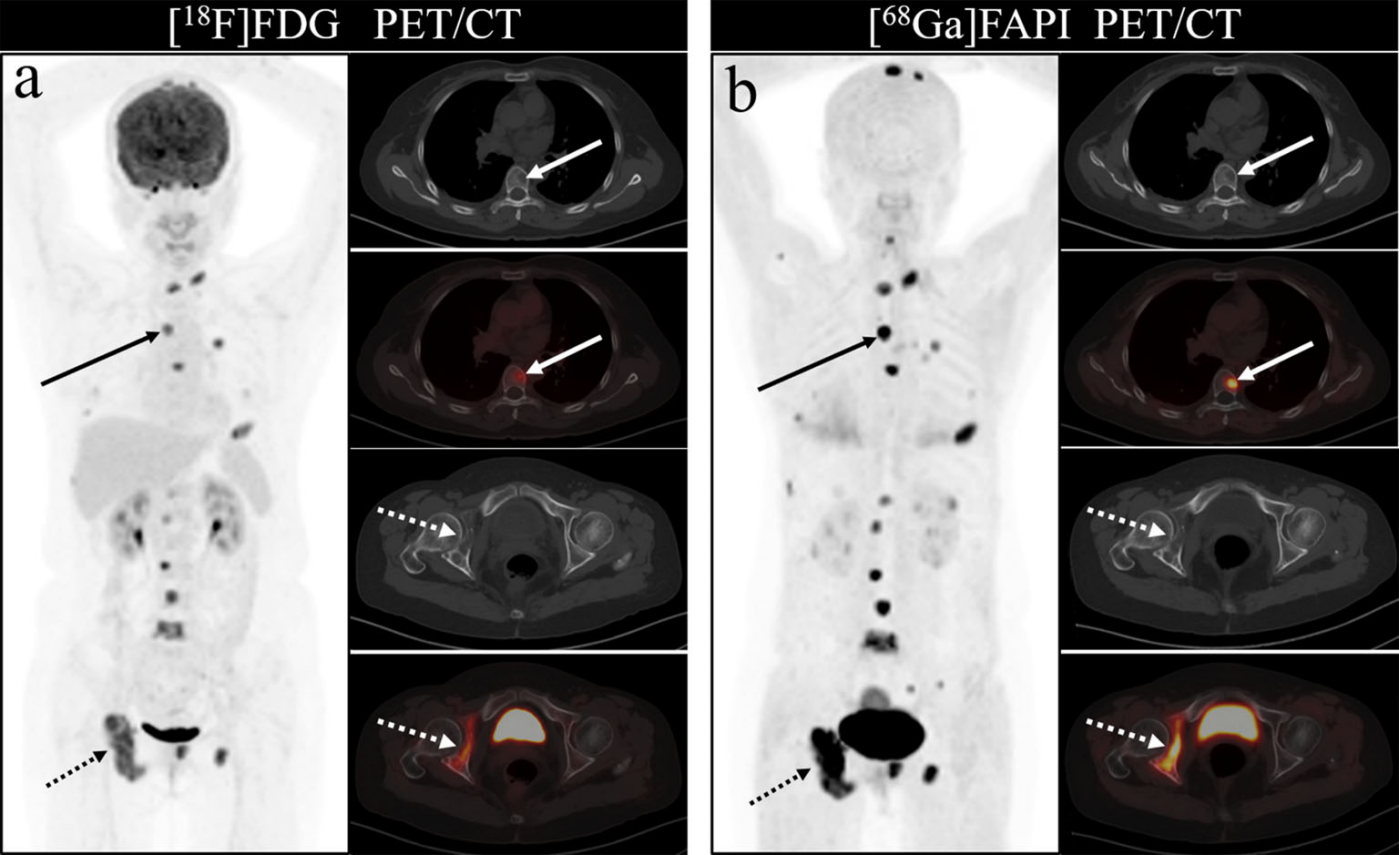
A 46-year-old woman with a newly diagnosed lung cancer underwent [^18^F]FDG and [^68^Ga]Ga-DOTA-FAPI-04 PET/CT **(A, B)**. The MIP images **(A, B)** of the [^18^F]FDG and [^68^Ga]Ga-DOTA-FAPI-04 PET/CT showed multiple bone lesions. On the selected axial images, [^18^F]FDG PET/CT **(A)** only showed mild to moderate [^18^F]FDG activities on thoracic vertebrae (arrows) and right acetabulum (dashed arrows), whereas [^68^Ga]Ga-DOTA-FAPI-04 PET/CT **(B)** showed intense [^68^Ga]Ga-DOTA-FAPI-04 uptakes in thoracic vertebrae (arrows) and right acetabulum (dashed arrows).

**Figure 4:**
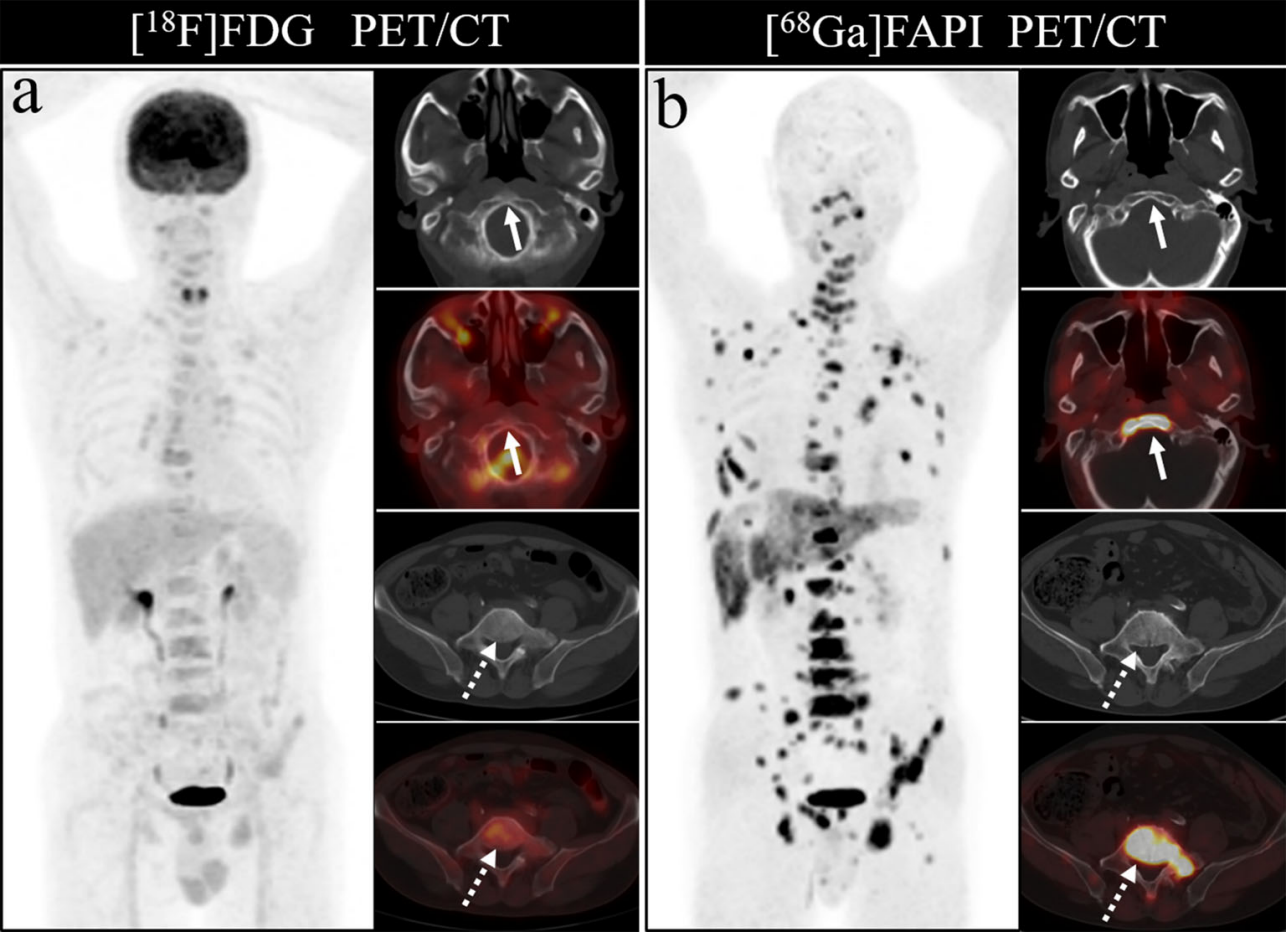
A 63-year-old man was received [^18^F]FDG and [^68^Ga]Ga-DOTA-FAPI-04 PET/CT **(A, B)** to evaluate the new diagnosed lung cancer. The MIP image **(A, B)** of [^18^F]FDG and [^68^Ga]Ga-DOTA-FAPI-04 PET/CT demonstrated multiple bone lesions. On the selected axial images, [^18^F]FDG PET/CT **(A)** only showed slight [^18^F]FDG activities in the clivus (arrows) and lumbar vertebral (dashed arrows). On the contrary, intense FAPI uptakes in the clivus (arrows) and lumbar vertebral (dashed arrows) were obviously observed on [^68^Ga]Ga-DOTA-FAPI-04 PET/CT.

Of these 109 lesions, 66 and 43 were characterized as osteolytic and osteoblastic bone metastases, respectively. All two types of the metastases were successfully detected by [^68^Ga]Ga-DOTA-FAPI-04 PET/CT imaging, while [^18^F]FDG PET/CT only detected 55/66 (83.3%) osteolytic and 34/43 (79.1%) osteoblastic metastases. The reason why [^68^Ga]Ga-DOTA-FAPI-04 has a higher detection rate for bone metastasis than [^18^F]FDG PET/CT may be due to the fact that [^68^Ga]Ga-DOTA-FAPI-04 radiotracer accumulation within bone metastases was significantly higher than that of [^18^F]FDG (n=109, median SUVmax, 9.1 *vs* 4.5; P< 0.01, respectively, **Figure 1**). In a subgroup analysis of radiotracer accumulation in osteolytic and osteoblastic metastases, [^68^Ga]Ga-DOTA-FAPI-04 accumulation was significantly higher than that of [^18^F]FDG in both osteolytic (n=66, median SUVmax, 10.6 *vs* 6.1; P < 0.01), and osteoblastic metastases (n=43, median SUVmax, 7.7 *vs* 3.7; P < 0.01, **Figure 2**). **Figures 3**, **4** offer an overview of the typical performance of [^68^Ga]Ga-DOTA-FAPI-04 and [^18^F]FDG PET/CT when evaluating osteolytic and osteoblastic metastases, respectively. In addition, [^68^Ga]Ga-DOTA-FAPI-04 or [^18^F]FDG uptakes was found to be significantly higher in the 66 osteolytic lesions relative to the 43 osteoblastic lesions.

**Figure 5:**
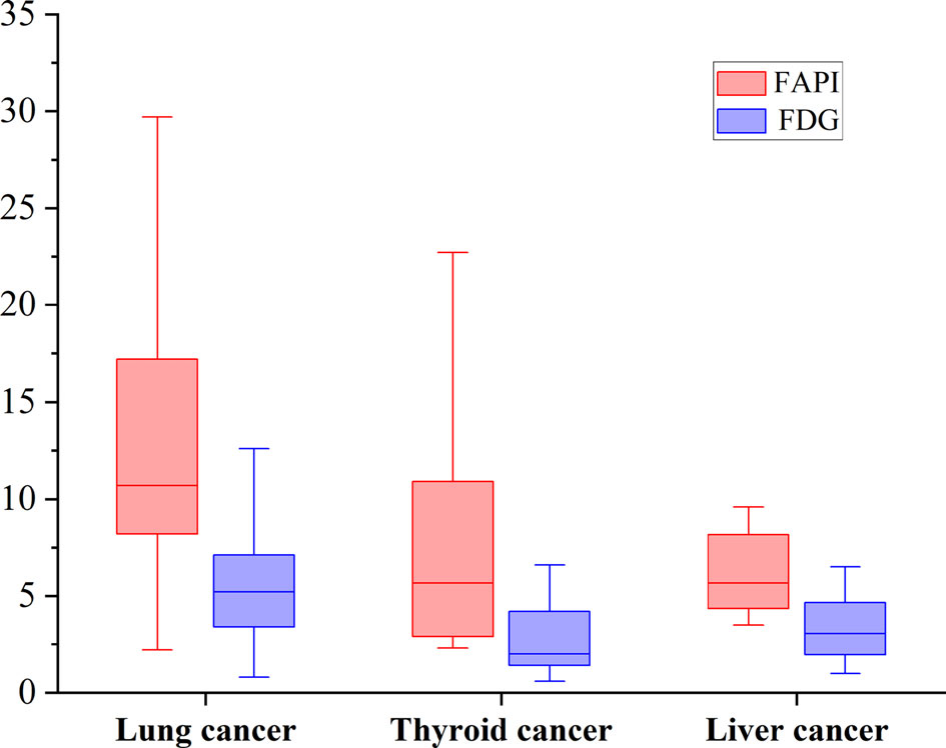
The SUVmax of FAPI and FDG in bone metastases from lung cancer, thyroid cancer and liver cancer. [^68^Ga]Ga-DOTA-FAPI-04 accumulation was significantly higher than that of [^18^F]FDG in bone metastases from lung cancer (n = 62, median SUVmax, 10.7 *vs* 5.2; P < 0.01), thyroid cancer (n = 18, median SUVmax, 5.65 *vs* 2.1; P < 0.01) and liver cancer (n = 12, median SUVmax, 5.65 *vs* 3.05; P < 0.01).

According to pathological types of primary tumors, we count the bone metastases from lung cancer (n = 11), thyroid cancer (n = 5) and liver cancer (n = 5). [^68^Ga]Ga-DOTA-FAPI-04 uptakes were significantly higher than that of [^18^F]FDG in bone metastases from lung cancer (n = 62, median SUVmax, 10.7 *vs* 5.2; P < 0.01), thyroid cancer (n = 18, median SUVmax, 5.65 *vs* 2.1; P < 0.01) and liver cancer (n = 12, median SUVmax, 5.65 *vs* 3.05; P < 0.01). [^68^Ga]Ga-DOTA-FAPI-04 or [^18^F]FDG uptakes was found to be significantly higher in lung cancer relative to the thyroid cancer and liver cancer, whereas [^68^Ga]Ga-DOTA-FAPI-04 or [^18^F]FDG uptakes between thyroid cancer and liver cancer is no significantly difference, **Figure 5**.

**Figure 6:**
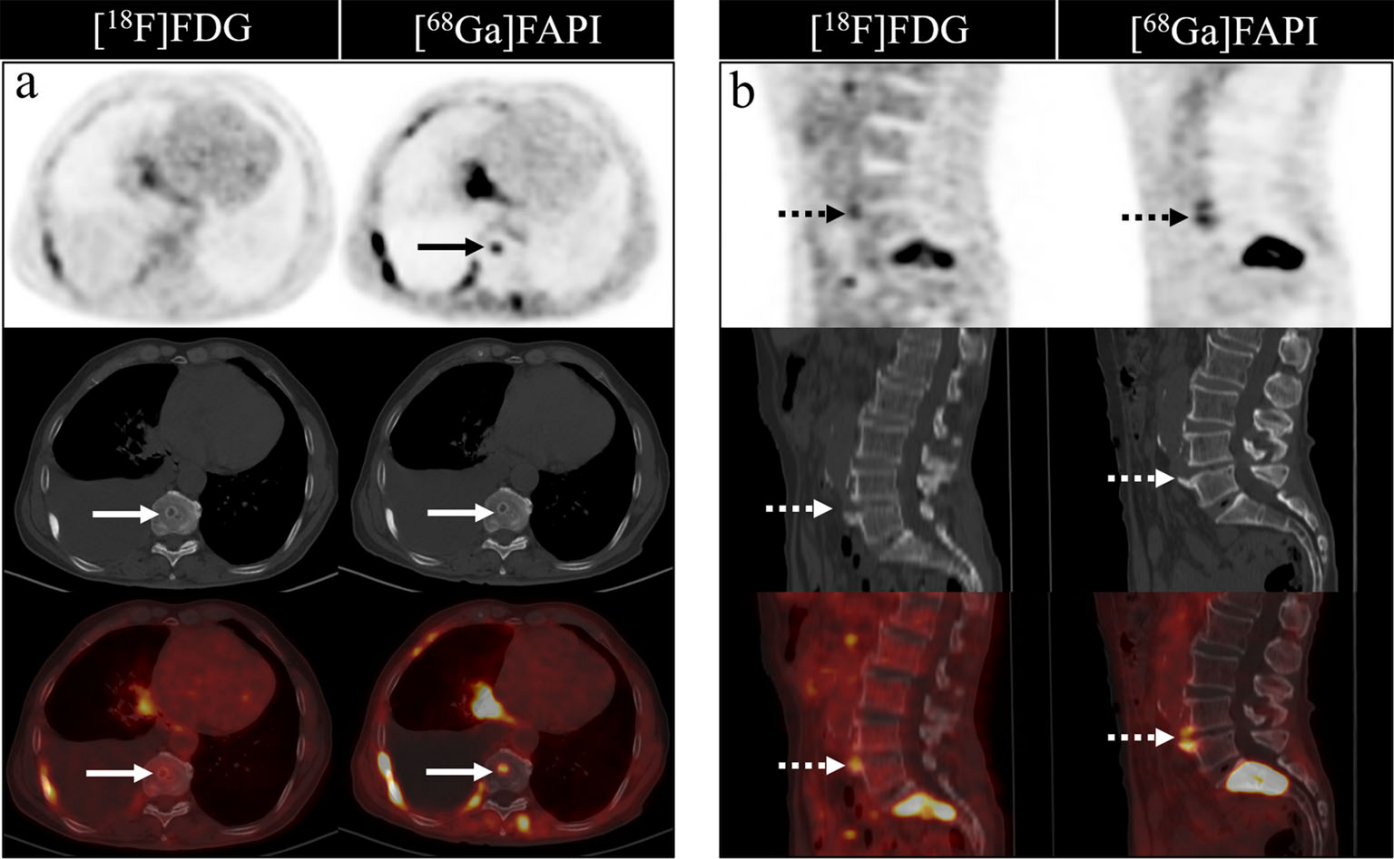
Characteristic findings of Schmorl Node **(A)** and degenerative osteophyte **(B)**. The Schmorl Node in the patient with lung cancer **(A)** shows tracer uptake on the [^68^Ga]Ga-DOTA-FAPI-04 PET/CT, while no uptake on the [^18^F]FDG PET/CT. The degenerative osteophyte in the patient with lung cancer **(B)** shows tracer accumulation on [^18^F]FDG and [^68^Ga]Ga-DOTA-FAPI-04 PET/CT.

[^68^Ga]Ga-DOTA-FAPI-04 PET/CT detected more false-positive lesions. Ten cases of false-positive uptakes were identified in patients undergoing [^68^Ga]Ga-DOTA-FAPI-04 PET/CT imaging, with benign lesions including degenerative osteophyte (4/10), arthritis (3/10), fractures (2/10) and Schmorl Nodes (1/10). In contrast, just five false-positive lesions were detected upon [^18^F]FDG PET/CT imaging, including arthritis (3/5), degenerative osteophyte (1/5), and fractures (1/5). **Figure 6** showed characteristic findings of false-positive lesions on [^18^F]FDG and [^68^Ga]Ga-DOTA-FAPI-04 PET/CT. False-positive uptake in Schmorl Nodes was observed on [^68^Ga]Ga-DOTA-FAPI-04 PET/CT, while no tracer accumulation was noted on [^18^F]FDG PET/CT. Begin degenerative osteophyte showed increased tracer uptakes on both [^18^F]FDG and [^68^Ga]Ga-DOTA-FAPI-04 PET/CT.

## Discussion

Bone metastases commonly arise as a consequence of the progression of many tumor types, leading to a range of potentially serious complications. As such, timely diagnosis is critical to the appropriate prognostic evaluation and treatment planning in cancer patients. Several imaging approaches have been developed in recent years and applied in an effort to detect bone metastases, but all of these methods are subject to certain limitations.

Integrated PET/CT imaging approaches to bone metastasis detection offer certain advantages over traditional imaging modalities, as they can better enable the identification of small metastatic lesions that would otherwise be overlooked. PET/CT approaches can additionally enable physicians to differentiate between metastases and benign degenerative lesions or fractures with a greater degree of accuracy, thus increasing diagnostic specificity and reducing the incidence of false-positive results (34). [^18^F]FDG PET/CT imaging has emerged in recent years as an efficacious imaging approach for the evaluation of bone metastases and other malignant lesions. By enabling the direct detection of increased glucose metabolism based upon differential [^18^F]FDG uptake, [^18^F]FDG PET/CT aid in the early identification of bone marrow involvement at a level of sensitivity likely to be overlooked by BS or CT imaging (35). Indeed, [^18^F]FDG PET/CT has been shown to enhance tumor staging accuracy and to thereby directly influence patient management. In one retrospective study, [^18^F]FDG PET/CT was shown to achieve a sensitivity value as high as 87% for the detection of bone marrow metastases, whereas BS achieved a sensitivity of just 29% (36). However, detecting metastases near sites of high [^18^F]FDG uptake such as the brain, liver, and gastrointestinal tract can be difficult, and this radiotracer may also yield false-positive results owing to altered metabolic activity associated with certain benign bone diseases (37, 38).

The recent development of quinolone-based [^68^Ga]Ga-labeled FAPI as a novel radiotracer for use in PET/CT applications has been shown to be associated with a number of advantages over [^18^F]FDG PET/CT in the context of tumor detection (25). For example, [^68^Ga]Ga-DOTA-FAPI-04 accumulates at high levels within tumors, and exhibits good pharmacokinetics and biochemical properties (25, 28). Several studies comparing the relative performance of these PET/CT imaging approaches have been conducted to date, with some having demonstrated that the majority of primary and metastatic lesions exhibit increased [^68^Ga]Ga-DOTA-FAPI-04 uptake relative to that of [^18^F]FDG (23, 28, 31). [^68^Ga]Ga-DOTA-FAPI-04 PET/CT can also yield better background signal levels in multiple tumor types, and there is some evidence that it can more reliably detect bone metastases as compared to [^18^F]FDG PET/CT. [^68^Ga]Ga-DOTA-FAPI-04 PET/CT may thus offer value as a tool for visualizing and monitoring bone metastases (23, 31, 39).

The present study was designed to compare the relative performance of [^68^Ga]Ga-DOTA-FAPI-04 and [^18^F]FDG PET/CT in patients with a range of cancer types harboring bone metastases. This analysis revealed [^68^Ga]Ga-DOTA-FAPI-04 PET/CT to be more sensitive than [^18^F]FDG PET/CT in this diagnostic context, with most metastatic bone lesions exhibiting increased uptake of [^68^Ga]Ga-DOTA-FAPI-04 relative to [^18^F]FDG, particularly for osteolytic lesions. [^68^Ga]Ga-DOTA-FAPI-04 PET/CT detected bone metastases in 30 patients, while [^18^F]FDG PET/CT only detected bone metastases in 26 patients. In addition, for the 23 primary lesions, [^68^Ga]Ga-DOTA-FAPI-04 PET/CT detected all of them, while 1 primary renal cancer was not visualized by [^18^F]FDG PET/CT. This result showed that [^68^Ga]Ga-DOTA-FAPI-04 can better detect and stage tumor patients compared to [^18^F]FDG PET/CT. As [^68^Ga]Ga-DOTA-FAPI-04 PET/CT detects bone metastases in a more sensitive manner at an earlier time point, it can provide good guidance for the treatment of tumor patients, expedite patient staging and thereby improve patient survival and quality of life.

Herein, [^68^Ga]Ga-DOTA-FAPI-04 PET/CT was able to successfully detected all bone metastases in analyzed patients, consistent with its excellent diagnostic performance in this context, whereas 20 bone metastatic lesions were missed by [^18^F]FDG PET/CT imaging. In line with our results, another prior study of patients with multiple tumor types found [^68^Ga]Ga-DOTA-FAPI-04 PET/CT to be more sensitive than [^18^F]FDG PET/CT in the detection of bone metastases (30). This enhanced sensitivity is likely at least partially attributable to the high uptake of [^68^Ga]Ga-DOTA-FAPI-04 by bone metastases. Indeed, we found that the SUVmax associated with [^68^Ga]Ga-DOTA-FAPI-04 was higher than that associated with [^18^F]FDG for most bone metastases. In their retrospective analysis, Chen et al. (23). similarly found that the SUVmax of [^68^Ga]Ga-FAPI-04 in bone metastases was significantly higher than that of [^18^F]FDG. Bone microenvironment is fertile soil for bone metastases. One of the important reasons is tumor cell-stromal cell interactions (40). This preferential [^68^Ga]Ga-DOTA-FAPI-04 accumulation is likely associated with the presence of CAFs in tumor stroma of bone metastases (41).

Physiological uptake of high levels of [^18^F]FDG is known to occur in the brain, liver, and gastrointestinal tract, limiting the utility of this radiotracer in the detection of metastases located proximal to these tissues. Certain benign lesions and conditions associated with hematopoietic cytokine stimulation, infections, fractures, benign bone lesions, and benign hematological diseases can cause abnormally increased [^18^F]FDG uptake, masking metastatic lesions or yielding false-positive results (35, 42, 43). In contrast, [^68^Ga]Ga-DOTA-FAPI-04 physiological uptake by the brain, liver, and gastrointestinal tract is significantly lower than that of [^18^F]FDG, yielding better contrast in tumor images (23, 29, 31).

**Figure 7:**
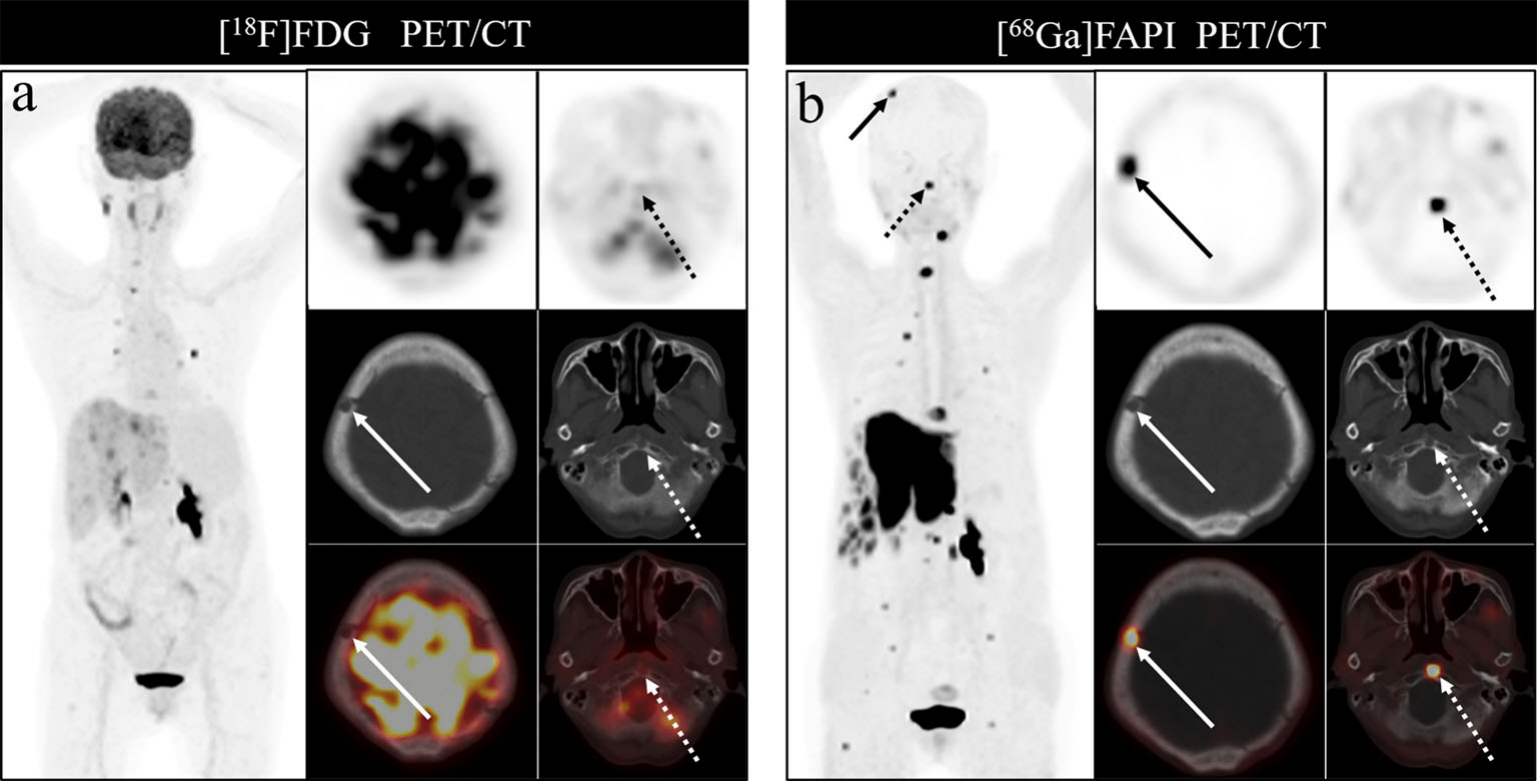
A 76-year-old woman underwent [^18^F]FDG and [^68^Ga]Ga-DOTA-FAPI-04 PET/CT **(A, B)** to assess possible recurrence of thyroid cancer. The MIP image **(A, B)** of [^18^F]FDG and [^68^Ga]Ga-DOTA-FAPI-04 PET/CT demonstrated multiple tracer activities bone and liver lesions. On the selected axial images, [^18^F]FDG PET/CT **(A)** showed limited [^18^F]FDG uptakes in the parietal bone (arrows) and clivus (dashed arrows), while [^68^Ga]Ga-DOTA-FAPI-04 PET/CT **(B)** showed intense [^68^Ga]Ga-DOTA-FAPI-04 uptakes in the parietal bone (arrows) and clivus (dashed arrows). In addition, the expression of [^68^Ga]Ga-DOTA-FAPI-04 in liver metastasis was significantly higher than that of [^18^F]FDG.

Bone metastases were frequently located in the vertebrae, ribs, and pelvis, in line with prior studies, likely owing to the presence of more metastatic deposits in these sites. [^68^Ga]Ga-DOTA-FAPI-04 PET/CT was able to detect metastases in all regions of the body, but [^18^F]FDG PET/CT had a poor detection rate for cranial, rib and long bone metastases. The detection rate of [^18^F]FDG PET/CT for cranial lesions was just 50%, whereas [^68^Ga]Ga-DOTA-FAPI-04 PET/CT detected all such lesions, likely owing to the masking effects of high intracranial glucose metabolism on [^18^F]FDG activity in cranial lesions. As intracranial [^68^Ga]Ga-DOTA-FAPI-04 uptake is limited, it can clearly resolve these lesions. **Figure 7** demonstrates the application of [^68^Ga]Ga-DOTA-FAPI-04 and [^18^F]FDG PET/CT for detecting cranial metastases. As such, [^68^Ga]Ga-DOTA-FAPI-04 may be better than [^18^F]FDG PET/CT as a tool for detecting bone metastases near the brain.

In line with prior studies, we found that [^18^F]FDG PET/CT exhibited a higher detection rate for osteolytic lesions relative to osteoblastic lesions. And we observe a significant trend towards increased [^18^F]FDG uptake in osteolytic lesions relative to osteoblastic metastases, which is consistent with prior studies (34). While [^68^Ga]Ga-DOTA-FAPI-04 PET/CT detected 100% of both osteolytic and osteoblastic bone metastases. Whether in osteolytic metastasis or osteoblastic metastases, [^68^Ga]Ga-DOTA-FAPI-04 PET/CT has a higher tracer accumulation relative to [^18^F]FDG, suggesting that [^68^Ga]Ga-DOTA-FAPI-04 PET/CT may have potential particularly advantageous in the detection of osteolytic and osteoblastic lesions.

Classification by pathological type of primary tumor, we found that [^68^Ga]Ga-DOTA-FAPI-04 has obviously higher accumulation of tracer agent than [^18^F]FDG, whether in lung cancer, thyroid cancer or liver cancer. The result showed that [^68^Ga]Ga-DOTA-FAPI-04 may have obvious advantages over [^18^F]FDG PET/CT for bone metastasis from different tumor subtypes. But there is still a need for more diverse tumor types to be included in further research. In addition, the [^68^Ga]Ga-DOTA-FAPI-04 and [^18^F]FDG activities in bone metastases of lung cancer were higher than that of thyroid cancer and liver cancer. The metabolic activity of bone metastases may be related to the pathological type of the primary tumor and the metabolic activity of the primary tumor, so further research is needed.

It is also important to note that multiple benign bone lesion types can cause an increase in [^68^Ga]Ga-DOTA-FAPI-04 expression. Consistent with this fact, we found that [^68^Ga]Ga-DOTA-FAPI-04 PET/CT imaging resulted in the detection of a greater number of false-positive bone lesions relative to the [^18^F]FDG PET/CT approach, suggesting that, while powerful, this former approach does not exhibit any advantage with respect to tumor specificity over [^18^F]FDG PET/CT imaging. Certain forms of bone lesions including degenerative osteophytes, arthritis, Schmorl nodes and fractures can result in false-positive [^68^Ga]Ga-DOTA-FAPI-04 uptakes, as has been reported previously (30, 44–47). In addition, increased [^68^Ga]Ga-DOTA-FAPI-04 uptake may also be associated with benign lesions associated with myelofibrosis (30) as a consequence of nonspecific fibrosis occurring therein (28). Careful CT image evaluation is thus critical to aid in the differentiation between true metastases and benign degenerative lesions or fractures, ensuring that physicians are able to accurately interpret [^68^Ga]Ga-DOTA-FAPI-04 PET/CT imaging result.

This study is subject to certain limitations. For one, this was a retrospective analysis of a relatively small patient group, and these results are thus susceptible to selection bias. Secondly, bone metastases were not confirmed *via* histological evaluation in the included patients owing to ethical and practical concerns regarding the biopsy of bone lesions in individuals with multiple suspected metastases. These metastases were instead confirmed based upon imaging examination results (BS, CT, MRI, or PET/CT) and clinical follow-up (physical signs and follow-up imaging examination). Finally, our study included a range of primary tumor types, introducing a degree of clinical heterogeneity that may have impacted the overall study results.

## Conclusion

In summary, [^68^Ga]Ga-DOTA-FAPI-04 PET/CT exhibits excellent diagnostic performance as a means of detecting bone metastases, performing in a manner superior to [^18^F]FDG PET/CT in this diagnostic context. [^68^Ga]Ga-DOTA-FAPI-04 radiotracer uptake by bone metastases was greater than that of [^18^F]FDG in this imaging context, suggesting that the use of the former tracer in the context of PET/CT imaging may be advantageous as a means of detecting bone metastases and guiding appropriate patient treatment. However, [^68^Ga]Ga-DOTA-FAPI-04 PET/CT imaging did lead to the detection of more false-positive lesions as compared to [^18^F]FDG PET/CT, reducing the overall specificity of this imaging modality and constraining its applicability as a means of detecting bone metastases. Overall, these results are only preliminary findings, and further large-scale prospective trials will be required to fully establish the value of [^68^Ga]Ga-DOTA-FAPI-04 PET/CT as a means of detecting bone metastases in patients with different underlying cancer types.

## Data Availability Statement 

The original contributions presented in the study are included in the article/supplementary material. Further inquiries can be directed to the corresponding author.

## Ethics Statement 

The studies involving human participants were reviewed and approved by Ethics Committee of Southwest Medical University. The patients/participants provided their written informed consent to participate in this study. Written informed consent was obtained from the individual(s) for the publication of any potentially identifiable images or data included in this article.

## Author Contributions

All authors listed have made a substantial, direct, and intellectual contribution to the work and approved it for publication.

## Conflict of Interest

The authors declare that the research was conducted in the absence of any commercial or financial relationships that could be construed as a potential conflict of interest.

## Publisher’s Note

All claims expressed in this article are solely those of the authors and do not necessarily represent those of their affiliated organizations, or those of the publisher, the editors and the reviewers. Any product that may be evaluated in this article, or claim that may be made by its manufacturer, is not guaranteed or endorsed by the publisher.
